# Carrier transfer in quasi-2D perovskite/MoS_2_ monolayer heterostructure

**DOI:** 10.1515/nanoph-2023-0570

**Published:** 2023-11-28

**Authors:** Chaochao Qin, Wenjing Wang, Jian Song, Zhaoyong Jiao, Shuhong Ma, Shuwen Zheng, Jicai Zhang, Guangrui Jia, Yuhai Jiang, Zhongpo Zhou

**Affiliations:** Henan Key Laboratory of Infrared Materials & Spectrum Measures and Applications, and School of Physics, Henan Normal University, Xinxiang 453007, China; School of Physical Science and Technology, ShanghaiTech University, Shanghai 201210, China

**Keywords:** quasi-2D perovskites, transition-metal dichalcogenides, heterostructure, transient absorption, carrier transfer

## Abstract

Two-dimensional layered semiconductors have attracted intense interest in recent years. The van der Waals coupling between the layers tolerates stacking various materials and establishing heterostructures with new characteristics for a wide range of optoelectronic applications. The interlayer exciton dynamics at the interface within the heterostructure are vitally important for the performance of the photodetector and photovoltaic device. Here, a heterostructure comprising two-dimensional organic-inorganic Ruddlesden–Popper perovskites and transition metal dichalcogenide monolayer was fabricated and its ultrafast charge separation processes were systematically studied by using femtosecond time-resolved transient absorption spectroscopy. Significant hole and electron transfer processes in the ps and fs magnitude at the interface of the heterostructure were observed by tuning pump wavelengths of the pump-probe geometries. The results emphasize the realization of the exciton devices based on semiconductor heterostructures of two-dimensional perovskite and transition metal dichalcogenide.

## 1 Introduction

Transitional metal dichalcogenides (TMDs) including MoS_2_, WS_2_, and WSe_2_ are promising semiconductor candidates for constructing potential novel ultrathin optoelectronic devices due to their unique physical properties [1–3]. Particularly, two-dimensional (2D) TMD monolayers hold strong photoluminescence (PL), high quantum efficiency over the direct optical bandgap ranging from visible to near-infrared regions, high carrier mobility, and extremely strong light–matter interactions, permitting them robust competitors in photodetectors and photovoltaics [4, 5]. Nevertheless, the high binding energy of excitons and the subordinate light absorbance from the atomically thin thickness of TMDs hinder their photo-sensing utilization [6]. Thus, exploring unprecedented 2D materials and exotic heterostructures with superior optical performance is truly anticipated for next-generation optoelectronic devices [7–9].

Quasi-2D organic–inorganic Ruddlesden–Popper perovskites (RPPs) have attracted tremendous concentration with large absorption coefficient, long carrier diffusion length, and good moisture stability, as one of the emerging popular materials for succeeding optoelectronic and photonic devices such as solar energy harvesting applications and light-emitting diodes with high power conversion efficiency in the recent years [10–13]. Quasi-2D RPPs adopt the formula of B_2_A_*n*−1_M_*n*_X_3*n*+1_, where B^+^ donates large organic cations such as phenemethylammonium (PEA^+^), A represents monovalent cation like Cs^+^, M is a divalent metal cation such as Pb^2+^, and X is a halide anion, and *n* defines the number of corner-sharing [MX_6_]^4−^ octahedral slabs between nearest two layers of B^+^ organic cations [14]. The quasi-2D perovskites with different diversity can be achieved through the incorporation of various bulky organic ligands, which ultimately show tunable optical and electronic properties [15–17]. Owing to the marvelous optical character and distinctive structure with commutative inorganic layer organic chain, 2D RPP can perform both as a photosensitive layer and an excellent charge-storage layer [18].

Coating 2D perovskite on top of a TMD monolayer to form a van der Waals (vdW) heterostructure provides an effective way to overcome the aforementioned drawbacks of TMDs and expand the functionalities in optoelectronics [19–23]. 2D perovskite/TMD heterostructures have been explored and utilized in photodetectors, photovoltaic devices, and optoelectronic memory [18, 24–29]. For instance, the detection performance has been enhanced several orders of magnitude compared to the isolated monolayer WS_2_ in 2D perovskite/WS_2_ heterostructure [24, 30]. The highly sensitive and ultrafast photoresponse at the near-infrared wavelength results from the interlayer transition of sub-band-gap photons and the fast separation of the photo-induced charges by the built-in potential in the 2D perovskite/MoS_2_ heterojunction [28]. Furthermore, the vis-infrared broadband nonvolatile optoelectronic memory based on few-layer MoS_2_/2D-RPP vdW heterojunction has been demonstrated that the 2D-RPP converts the initial n-type MoS_2_ into p-type and facilitates hole transfer between them [31]. However, the excitonic properties and excitation transfer mechanism (charge or energy transfer) of quasi-2D hybrid perovskite/TMD monolayer heterostructures are still to be elucidated [32, 33]. Until now, the reports are mostly limited to PEA and BA containing *n* = 1 2D lead iodide perovskite for 2D perovskite/TMD heterostructures, and even these investigations are far from being conclusive [34, 35]. For example, it is still lacking for a systematic study of the selection of organic spacers with different chain lengths to tune the excitation transfer mechanism where the charge or energy transfer is highly sensitive to the distance between the perovskite donor and the TMD acceptor in the 2D perovskite/TMD heterostructure.

In this work, we investigate the interfacial exciton dynamics by constructing heterostructures with MoS_2_ monolayer and the quasi-2D RP perovskite (TEA)_2_Cs_2_Pb_3_Br_10_ where TEA is S-contained thiophene-2-ethylamine (C_6_H_9_NS). The transfer of the photoexcited carriers in the heterostructure interface is systematically studied by employing ultrafast transient absorption (TA) spectroscopy. Importantly, we directly observed the hole-transfer process during 28.3 ps by selectively stimulating electrons in MoS_2_ and probing exciton states in the (TEA)_2_Cs_2_Pb_3_Br_10_. The process of electron transfer is observed within 0.12 ps in the heterostructure. Interestingly, the spatially separated electron and hole pairs form interlayer excitons that possess longer lifetime than their counterparts. This work gives a comprehensive understanding of the ultrafast exciton dynamics in (TEA)_2_Cs_2_Pb_3_Br_10_/MoS_2_ heterostructure.

## 2 Results and discussion

**Figure 1:**
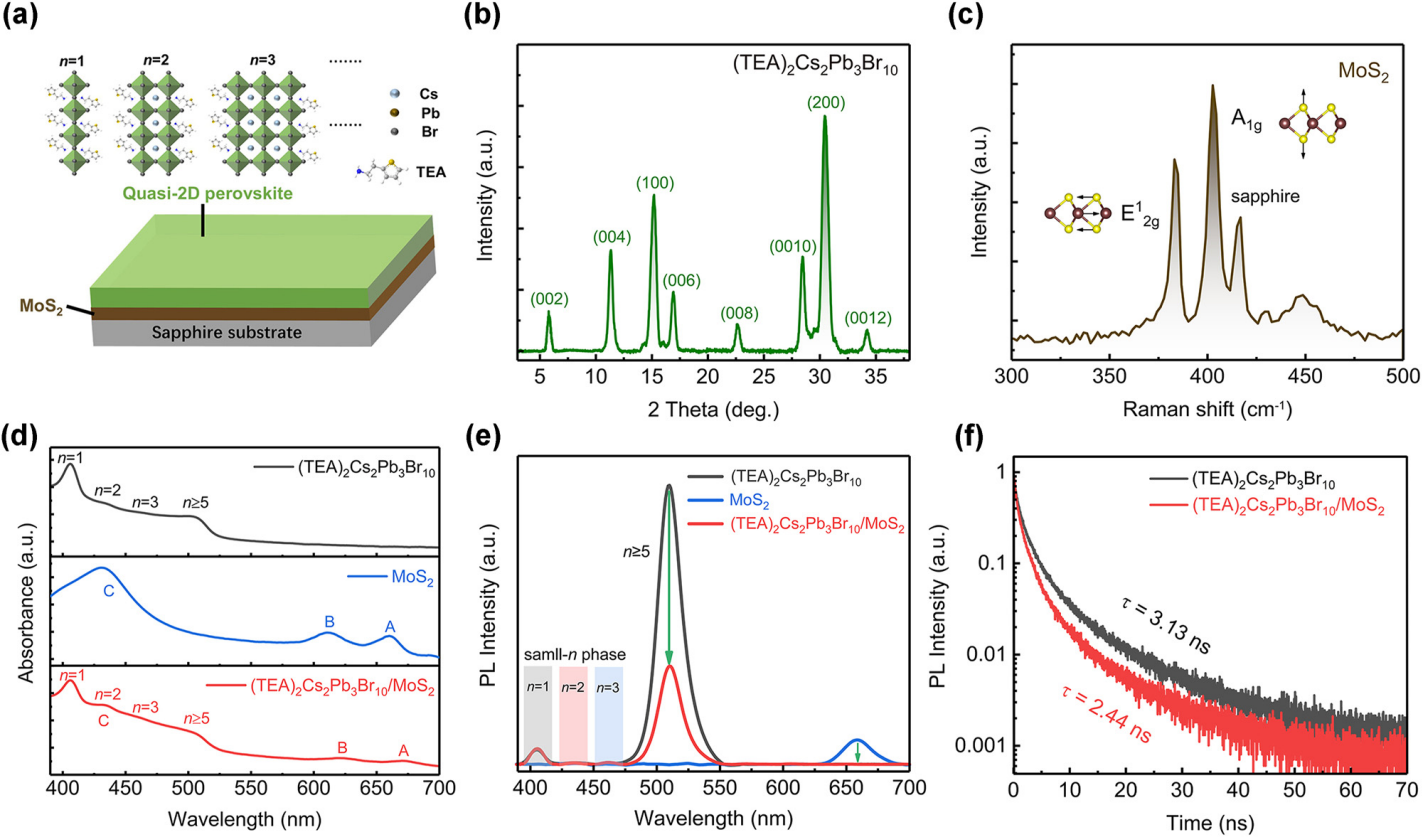
Structure and optical properties. (a) The schematic of (TEA)_2_Cs_2_Pb_3_Br_10_/MoS_2_ heterostructure. (b) XRD patterns for (TEA)_2_Cs_2_Pb_3_Br_10_ film. (c) Raman spectra of an isolated monolayer MoS_2_ grown on a sapphire substrate under 532 nm excitation. (d) Absorption spectra of (TEA)_2_Cs_2_Pb_3_Br_10_, monolayer MoS_2_, and the heterostructure. (e) PL spectra of (TEA)_2_Cs_2_Pb_3_Br_10_, monolayer MoS_2,_ and the heterostructure under 375 nm laser excitation at room temperature. (f) The PL decay curve between the individual (TEA)_2_Cs_2_Pb_3_Br_10_ and heterostructure is compared at 510 nm, respectively.

The schematic illustration of a (TEA)_2_Cs_2_Pb_3_Br_10_/MoS_2_ heterostructure is shown in Figure 1a where quasi-2D (TEA)_2_Cs_2_Pb_3_Br_10_ and MoS_2_ monolayer are stacked on a sapphire substrate. The quasi-2D RPP can be considered as the insertion of organic spacers TEA into the 3D perovskite CsPbBr_3_ framework [36]. The alternating organic sheets and inorganic slabs with different energy bandgaps (*E*_g_) as potential barriers and wells, respectively, generate an ordered self-assembly multiple quantum wells (QWs) structure [37]. The X-ray diffraction (XRD) pattern of the film is shown in Figure 1b. The pristine quasi-2D RPP film exhibits characteristic peaks at 5.8°, 11.3°, 17.0°, 22.7°, 28.5°, and 34.2°, corresponding to the (002), (004), (006), (008), (0010) and (0012) planes of the 2D perovskite [38]. The characteristic peaks at 15.1° and 30.5° represent the crystallographic planes of (100) and (200) of the 3D cubic CsPbBr_3_, respectively, which suggests that CsPbBr_3_ nanocrystals appear [37], although the thiophene-based TEA^+^ organic cations with a *π*–*π* conjugate structure can constrain the growth of the 3D CsPbBr_3_ grains and reduce Pb^2+^ ion defects [39, 40]. Thus the small/intermediate-*n* and large-*n* phases coexist in the (TEA)_2_Cs_2_Pb_3_Br_10_ film. The Raman spectra of the MoS_2_ exhibit two distinct peaks at 384 cm^−1^ and 403 cm^−1^, corresponding to the in-plane mode (*E*_2g_^1^) and extra-surface mode (*A*_1_^g^), respectively (Figure 1c) [41]. The energy difference of 19 cm^−1^ between the two Raman active modes matched well with the reported values [42, 43], which confirm the monolayer nature of the isolated MoS_2_. Besides, the phonon signal of sapphire is also reflected in the Raman spectra. Figure 1d shows the steady-state absorption spectra of quasi-2D RPP, MoS_2_ monolayer, and the heterostructure, respectively. The exciton absorption peaks at 406 nm (*n* = 1), 434 nm (*n* = 2), and 464 nm (*n* = 3), and the bandgap absorption around 510 nm (*n* ≥ 5) are observed, indicating that the as-prepared quasi-2D RPP film is composed of both small-*n* and large-*n* phases. For the MoS_2_ monolayer, there are three exciton absorption peaks at 660 nm (A-exciton), 611 nm (B-exciton), and 430 nm (C-exciton), respectively, corresponding to the direct exciton transitions from the valence band (VB) to the conduction band (CB) at K-point and Γ-point in the Brillouin zone [44]. The A and B excitons are resulted from the valence band splitting (Δ) which is induced by spin–orbit coupling, and the C-exciton has strong photon absorption characteristic in the band nesting region [41]. The absorption peaks of the heterostructure are at 405 nm, 434 nm, 464 nm, 510 nm 620 nm, and 670 nm, as a signal superposition of quasi-2D RPP and MoS_2_ monolayer. However, the monolayer MoS_2_ shows an obvious red shift of the absorption peaks for A-exciton and B-exciton, indicating that the band structure of the MoS_2_ monolayer has changed due to the interlayer coupling [45, 46].

Figure 1e shows PL spectra of quasi-2D RPP, monolayer MoS_2_, and the heterostructure with photoexcitation at 375 nm. The quasi-2D RPP film exhibits a strong emission peak at around 510 nm from the *n* ≥ 5 phases, a weak peak at 405 nm related to the *n* = 1 phase, and two tiny emission peaks at 436 nm and 463 nm attributed to the *n* = 2 and 3 phases, respectively [47]. The PL peak located at 660 nm is from the A-exciton of monolayer MoS_2_. In (TEA)_2_Cs_2_Pb_3_Br_10_/MoS_2_ composite, the PL intensities for the large-*n* phases of the RPP and the monolayer MoS_2_ are strongly quenched by ∼65 % and 100 %, respectively. No obvious emission quenching or enhancement is observed in the small-*n* phase. It indicates that there is charge transfer in the heterostructure instead of energy transfer which would cause a PL enhancement in the (TEA)_2_Cs_2_Pb_3_Br_10_ or MoS_2_ side. Figure 1f shows the time-resolved photoluminescence decay curves of quasi-2D RPP and the heterostructure, respectively. The TRPL curves are fitted by a biexponential function and the average lifetime of exciton radiative recombination is 3.13 ns and 2.44 ns for the RPP and the heterostructure, respectively, comparable to the reported values [48, 49]. The fitting details on the time-resolved PL (TRPL) curves are shown in Table S1. Compared with pristine RPP, the presence of MoS_2_ leads to a shorter exciton lifetime of the RPP in the heterostructure. These results indicate ultrafast interfacial charge transfer between (TEA)_2_Cs_2_Pb_3_Br_10_ and MoS_2_, which leads to the formation of interlayer indirect exciton in the heterostructure. The formation process of indirect excitons in the heterostructure can be regarded as an additional non-radiative exciton relaxation channel, and the indirect exciton formation process competes with the exciton radiative recombination process in RPP. The indirect exciton dissipates its energy through lattice vibration or other channels [50, 51].

**Figure 2:**
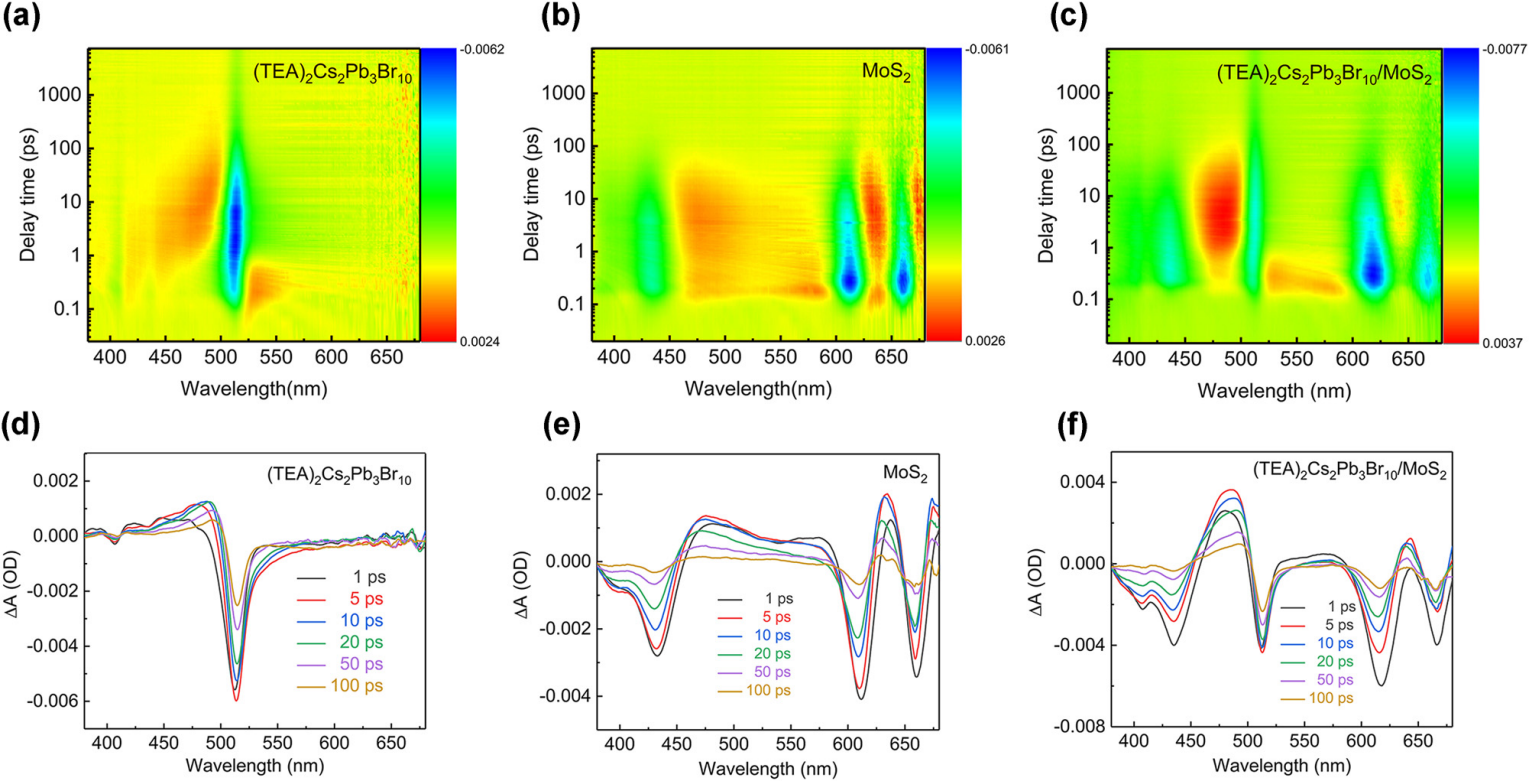
TA spectra. (a)–(c) Vis-pseudocolor representation of TA spectra with 365 nm excitation. (d)–(f) TA spectra at selected time scales.

To understand the exciton dynamics and the interfacial charge transfer in the heterostructure, we performed a microscopic transient absorption (TA) measurement in the range of 380 nm–680 nm with a pump wavelength of 365 nm and an excitation intensity of 45 μJ/cm^2^. As shown in Figure 2a–d, four ground-state bleaching (GSB) bands peaked at 405 nm, 434 nm, 464 nm, and 511 nm, agree well with the peaks in the absorption spectra of the quasi-2D RPP thin film, which are attributed to *n* = 1, 2, 3, and *n* ≥ 5 (*n* = ∞) phases. The GSB signal peaked at 510 nm for *n* ≥ 5 phases reaches the maximum at 2.09 ps, and the GSB lifetime is long up to 1 ns in the perovskite. For monolayer MoS_2_, the GSB peaks are located at 430 nm, 613 nm, and 660 nm. The GSB peaks of the heterostructure are the sum of the bleached signals for individual RPP and MoS_2_, consistent with the absorption spectrum in Figure 1d. For the A-exciton, the GSB of MoS_2_ monolayer and (TEA)_2_Cs_2_Pb_3_Br_10_/MoS_2_ heterostructure reaches the maximum at about 0.21 ps and 0.34 ps, respectively.

To explore the charge-transfer process and the exciton formation in the heterostructure after photoexcitation, we measured a series of ultrafast TA spectra by using specific resonant optical excitations. The excitation wavelength of 660 nm is used to excite the A-exciton of MoS_2_ monolayer (1.88 eV) to probe the hole transfer from MoS_2_ to (TEA)_2_Cs_2_Pb_3_Br_10_. The excitation wavelength of 510 nm (2.43 eV) is selected for (TEA)_2_Cs_2_Pb_3_Br_10_ to detect electron transfer from (TEA)_2_Cs_2_Pb_3_Br_10_ to MoS_2_. The excitation wavelength of 365 nm (3.4 eV) is employed for the hole transfer and electron transfer in the heterostructure interface.

### 2.1 Hole transfer process

**Figure 3:**
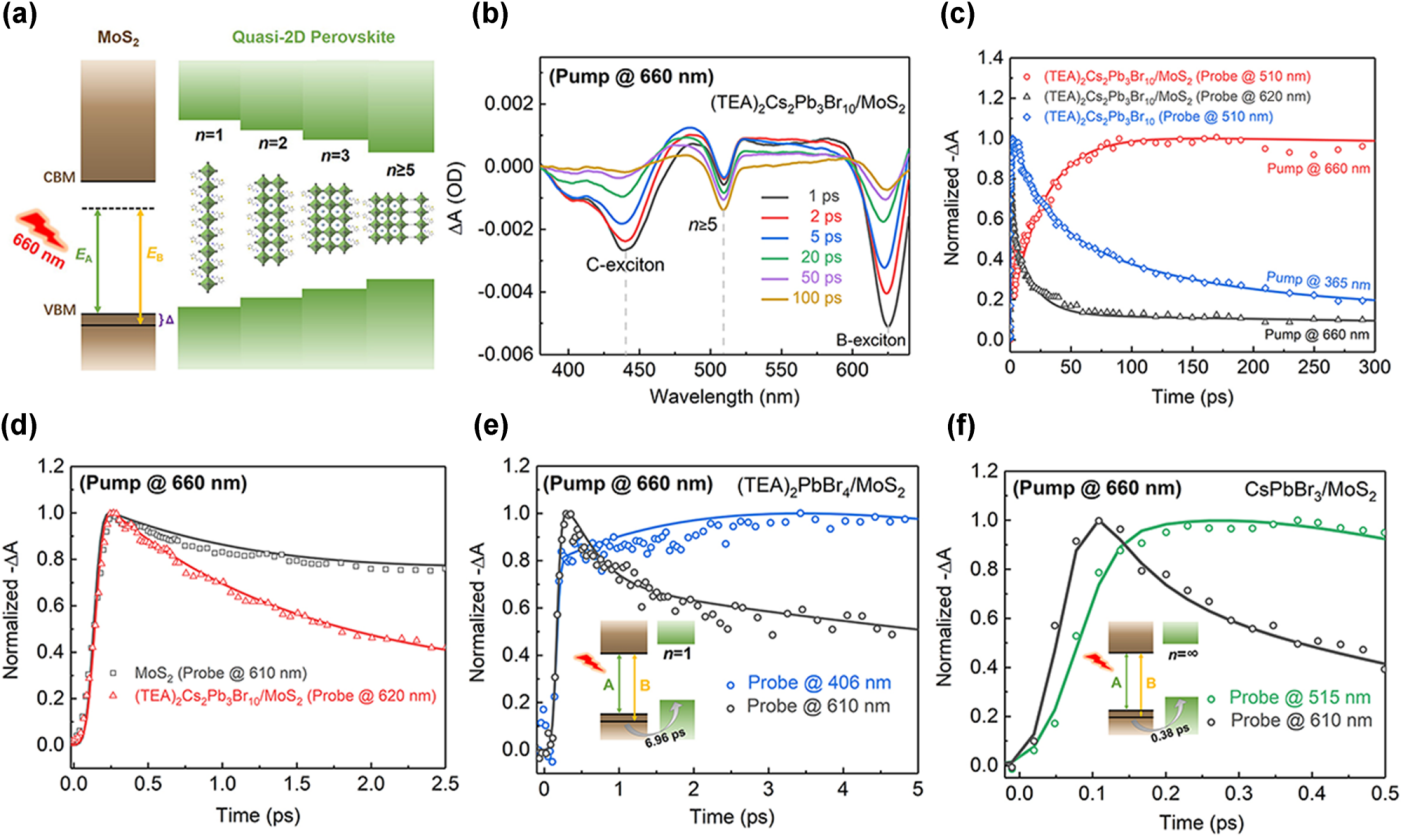
Dynamics of the hole transfer process. (a) Schematics of band alignment of the (TEA)_2_Cs_2_Pb_3_Br_10_/MoS_2_ heterostructure according to Refs. [44, 57], where CBM, VBM, *E*_A_, and *E*_B_ stand for CB minimum, VB maximum, the energy of exciton A and B. (b) TA spectra at selected timescales under 660 nm excitation. (c) TA kinetic curves at 510 nm and 620 nm for (TEA)_2_Cs_2_Pb_3_Br_10_/MoS_2_ under 660 nm excitation and isolated (TEA)_2_Cs_2_Pb_3_Br_10_ at 510 nm under 365 nm excitation. (d) TA kinetic curves at 610 nm and 620 nm for monolayer MoS_2_ and (TEA)_2_Cs_2_Pb_3_Br_10_/MoS_2_, respectively. (e) TA kinetic curves of (TEA)_2_PbBr_4_/MoS_2_ at 406 nm and 610 nm, respectively. (f) TA kinetic curves of CsPbBr_3_/MoS_2_ at 515 nm and 610 nm, respectively.

The electron transfer process from monolayer MoS_2_ to quasi-2D RPP was examined under excitation at 660 nm (1.88 eV) with 90 μJ/cm^2^ to near-resonantly excite the A-exciton transition of MoS_2_ (Figure 3a). Figure 3b shows the TA spectra at selected timescales for (TEA)_2_Cs_2_Pb_3_Br_10_/MoS_2_ heterostructure under 660 nm excitation. In the heterostructure, the GSB peak at 620 nm, 510 nm, and 440 nm correspond to the MoS_2_ B-exciton, the RPP *n* ≥ 5, and the MoS_2_ C-exciton, respectively. The signals of B and C excitons are formed based on the state-filling effect and nesting effect [51]. As 1.88 eV is less than the bandgap energy of (TEA)_2_Cs_2_Pb_3_Br_10_ which only excites the MoS_2_ layer, the GSB of the RPP in heterostructure can only be attributed to the transfer of photogenerated holes. The kinetic curves of the heterostructure at 510 nm and 620 nm under excitation of 660 nm, and the individual perovskite at 510 nm excited with 365 nm are exhibited in Figure 3c. The decay for the kinetic curve of *n* ≥ 5 phases in the heterostructure excited by 660 nm is significantly slower than that of the individual perovskite excited by 365 nm, further proving that the *n* ≥ 5 signal is generated by the transfer of photogenerated holes. Besides, the rapid decay of the B-exciton of MoS_2_ is followed by the slow formation of the large-*n* phase signal, indicating that the holes in the MoS_2_ are slowly injected into the perovskite.

Figure 3d shows the time evolution dynamics of B-exciton in monolayer MoS_2_ and heterostructure at 610 nm and 620 nm, respectively. To elucidate the charge transfer in the heterostructure, a generalized sequential kinetic model with three components is utilized to describe the spectral evolution. The TA kinetic curves in Figure 3d are fitted with the triple-exponential equation,

(1) $$\Delta A\left(t\right)=a_{1}e^{-t/\tau_{1}}+a_{2}e^{-t/\tau_{2}}-ce^{-t/\tau_{et}}$$

where *a*_1_, *a*_2_, and *c* stand for amplitudes, *τ*_1_, and *τ*_2_ denote decay time constants, and *τ*_*et*_ is the formation time constant [52]. The fitting parameters are shown in Tables S2–5. The time constant *τ*_1_ for the fast decay component and *τ*_2_ for the slow decay component are obtained at about 0.71 ps (0.38 ps) and 20.2 ps (12.5 ps) for B-exciton of MoS_2_ (heterostructure), ascribing to the cooling time of the holes and the lifetime of the carriers, respectively. Similarly, for the dynamic curve of the C-exciton (Figure S2), the time constant *τ*_1_ and *τ*_2_ are obtained at about 1.54 ps and 21.8 ps for MoS_2_ in the heterostructure, shorter than the hole lifetime of C-exciton in monolayer MoS_2_ (*τ*_1_ = 6.89 ps, *τ*_2_ = 42.8). The rise time of the RPP signal in the heterostructure directly designates the hole transfer across the interface, because this signal exists only after hole transfer. This hole transfer time of 28.3 ps is obtained by fitting the formation time in perovskite of the heterojunction, indicating that holes transferred from the MoS_2_ layer to the RPP layer within 28.3 ps after photo-excitation. This hole transfer time seems longer than that of other van der Waals heterostructure systems, which are usually in the order of picosecond due to the weak exciton-phonon coupling [53]. Besides, one of the factors that affect the charge transfer speed in atomic thin heterostructures is the distance between the two heterogeneous layers [54]. For (TEA)_2_Cs_2_Pb_3_Br_10_ perovskite, the small-*n* phases tend to stay at the bottom (near the MoS_2_ layer), while the large-*n* phases tend to form near the surface (away from the MoS_2_ layer), making the charge transfer time longer.

The control experiments on the charge transfer in the heterostructures were conducted by utilizing perovskites with different *n* values of 1 and infinite, i.e. (TEA)_2_PbBr_4_, and CsPbBr_3_, respectively. The static-state absorption peaks of (TEA)_2_PbBr_4_ and CsPbBr_3_ of the RPP/MoS_2_ heterostructures are located at 406 nm and 515 nm, respectively (Figure S3). Figure 3e and f show the dynamic curves for *n* = 1 (406 nm) perovskite and MoS_2_ B-exciton (610 nm) in the (TEA)_2_PbBr_4_/MoS_2_, as well as the dynamic curves for 3D CsPbBr_3_ (515 nm) and MoS_2_ B-exciton (610 nm) in CsPbBr_3_/MoS_2_, pumped with 660 nm wavelength, respectively. The rising and decaying process of the perovskite kinetics can be fitted with a triple-exponential function, which results in a hole transfer time of 0.38 ps for CsPbBr_3_/MoS_2_, shorter than the value of 6.96 ps for (TEA)_2_PbBr_4_/MoS_2_ and 28.3 ps of (TEA)_2_Cs_2_Pb_3_Br_10_/MoS_2_. It confirms that the hole transfer time from hundreds of femtoseconds to tens of picoseconds is relative to how close 3D perovskite to the MoS_2_ layer.

The TA measurements of (TEA)_2_Cs_2_Pb_3_Br_10_/MoS_2_ heterostructure establish unambiguously that optical excitation in MoS_2_ leads to the signal of exciton transitions in RPP. This provides direct evidence of efficient charge separation in the photoexcited heterostructure. Electron-hole pairs are initially created in the MoS_2_ layer, but the holes transfer to the RPP layer, while electrons stay in the MoS_2_ layer. Consequently, PL from MoS_2_ exciton resonances will be strongly quenched, as observed in Figure 1e.

### 2.2 Electron transfer process

**Figure 4:**
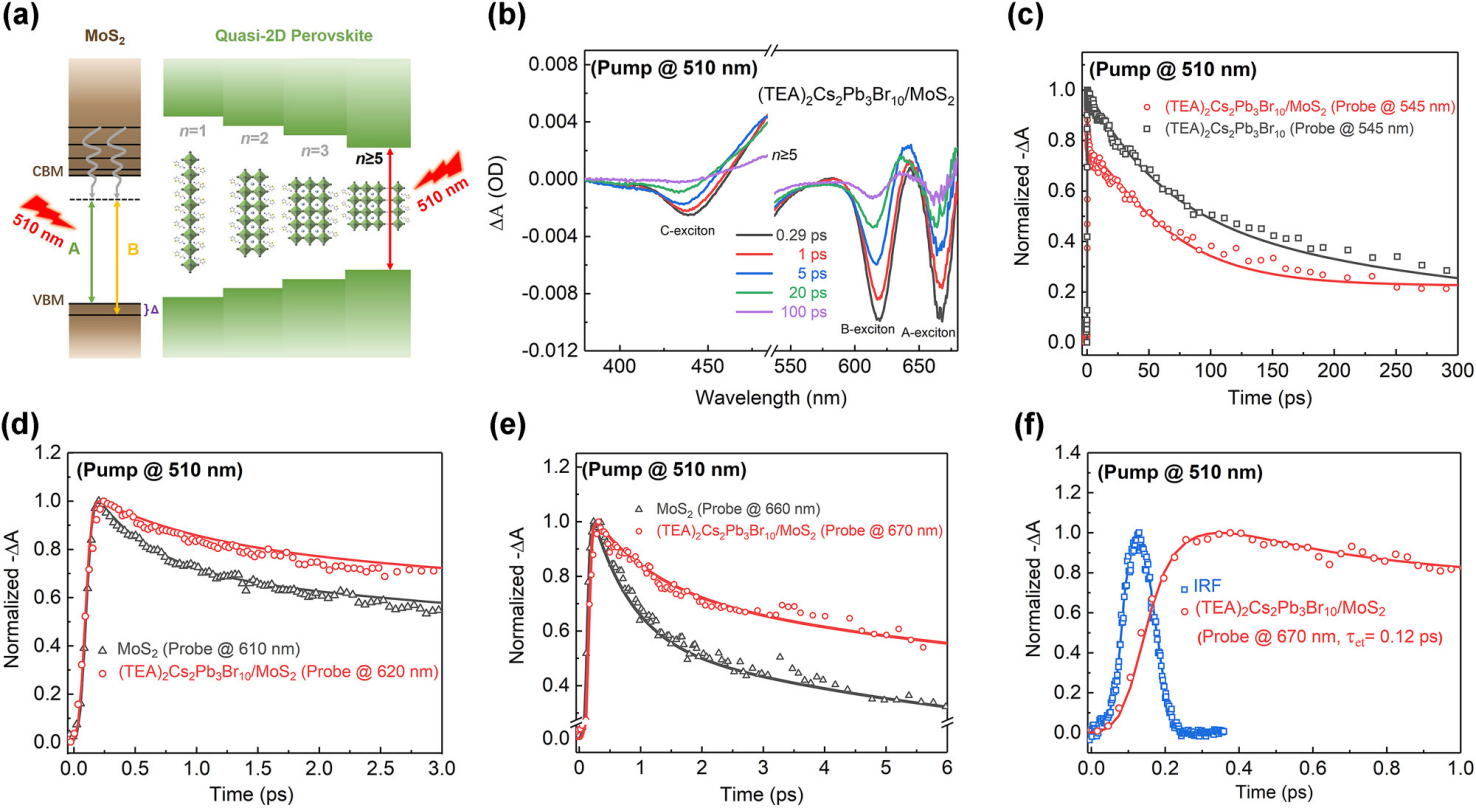
Dynamics of the electron transfer process. (a) Schematics of band alignment of the heterostructure and the pump-probe configuration. (b) TA spectra at selected timescales. (c) TA kinetic curves at 545 nm for neat RPP and heterostructure. (d) TA kinetic curves at 610 nm and 620 nm of B-exciton in neat MoS_2_ and heterostructure, respectively. (e) TA kinetic curves at 660 nm and 670 nm of MoS_2_ A-exciton and heterostructure, respectively. (f) Instrument response function and GSB dynamic curve of the heterostructure within 1.0 ps.

To further unravel the interfacial electron transfer process, we performed TA measurements by exciting quasi-2D RPP with a 510 nm (2.43 eV) pump pulse with 75 μJ/cm^2^, and a 660 nm (1.88 eV) probe pulse was tuned to the exciton resonance of MoS_2_ (Figure 4a). Figure 4b shows the TA spectra at selected timescales for the heterostructure with 510 nm excitation. The GSB signals at 670 nm, 620 nm, and 440 nm are attributed to the A, B, and C exciton of MoS_2_, respectively. The signal of *n* ≥ 5 phase is located near 510 nm (not shown here due to the overlap with the pump light), and there are no obvious GSB peaks of small-*n* phase RPP that cannot be excited by 510 nm wavelength. The type-II electronic band alignment of (TEA)_2_Cs_3_Pb_3_Br_10_ 2D RPP and MoS_2_ monolayer can facilitate interlayer charge transfer, where photogenerated electrons in RPP transfer and take over the conduction band of MoS_2_ and holes remain at the valence band of RPP. The spatially separated electron-hole pairs form interlayer excitons. The electrons in the interlayer excitons subsequently relax to the band minima and the A and B excited states. Since the A and B excitons of MoS_2_ can also be excited by the 510 nm pump, the hole transfer cannot be ruled out in this scenario. However, the generation efficiency for the hole carries with the 510 nm non-resonant excitation in MoS_2_ is lower than that of the direct transition between exciton states [55]. Consequently, the A and B excitons originate mainly from the photoinduced electrons in large-*n* phase RPP rather than the intralayer excited states of MoS_2_ in the heterostructure.

Figure 4c displays the normalized TA dynamic curves detected at 545 nm of neat (TEA)_2_Cs_2_Pb_3_Br_10_ and the MoS_2_/(TEA)_2_Cs_2_Pb_3_Br_10_ heterostructure, respectively. A rapid decay for the heterostructure is observed, faster than that of the (TEA)_2_Cs_2_Pb_3_Br_10_, verifying that the hot electrons in perovskite transfer to MoS_2_. Figure 4d displays the normalized TA dynamic curves of monolayer MoS_2_ and the heterostructure detected at 610 nm and 620 nm, respectively. Figure 4e shows the normalized TA dynamic curves of monolayer MoS_2_ and the heterostructure detected at 660 nm and 670 nm, respectively. The signal reaches a maximum immediately after the photo-excitation for each of the four samples in Figure 4d and e. In particular, a sluggish decay of the heterostructure is observed, longer than the steep decay of the monolayer MoS_2_, suggesting the spatial separation of the electrons and holes in the heterostructure. Figure 4f shows the instrument response function (IRF) and the time-evolution dynamics of the normalized GSB signal for the heterostructure with a timescale of 1 ps. The rising signal at 670 nm reaches the maximum at 347 fs, almost 3.5 times larger than the instrument response time, indicating that the electrons of the (TEA)_2_Cs_2_Pb_3_Br_10_ pass rapidly to the MoS_2_ through the vdW interface after the selective excitation. It is consistent with the previous results of PL quenching of perovskite. The fast decay constant *τ*_1_ and the slow decay constant *τ*_2_ for A-exciton of monolayer MoS_2_ are obtained to be 0.26 ps and 6.92 ps, respectively. By fitting the TA curve with the tri-exponential equation for the heterostructure, the fast decay constant *τ*_1_, slow decay constant *τ*_2,_ and formation time constant *τ*_*et*_ are estimated at 1.08 ps, 13.70 ps, and 0.12 ps, respectively. The carrier lifetime in the heterostructure is approximately 2 times the exciton lifetime in monolayer MoS_2_ owing to charge separation. The formation time of 0.12 ps represents the electron transfer process from the perovskite layer to MoS_2_. The electrons in MoS_2_ and holes in perovskite form interlayer excitons. The lifetime of the slow decay of the A-exciton (13.70 ps) represents the recombination process of interlayer excitons. Due to the interfacial coupling between perovskite and MoS_2_, the lifetime of the interfacial excitons in heterostructure is longer than that of the intralayer excitons in monolayer MoS_2_.

### 2.3 Both hole transfer and electron transfer

**Figure 5:**
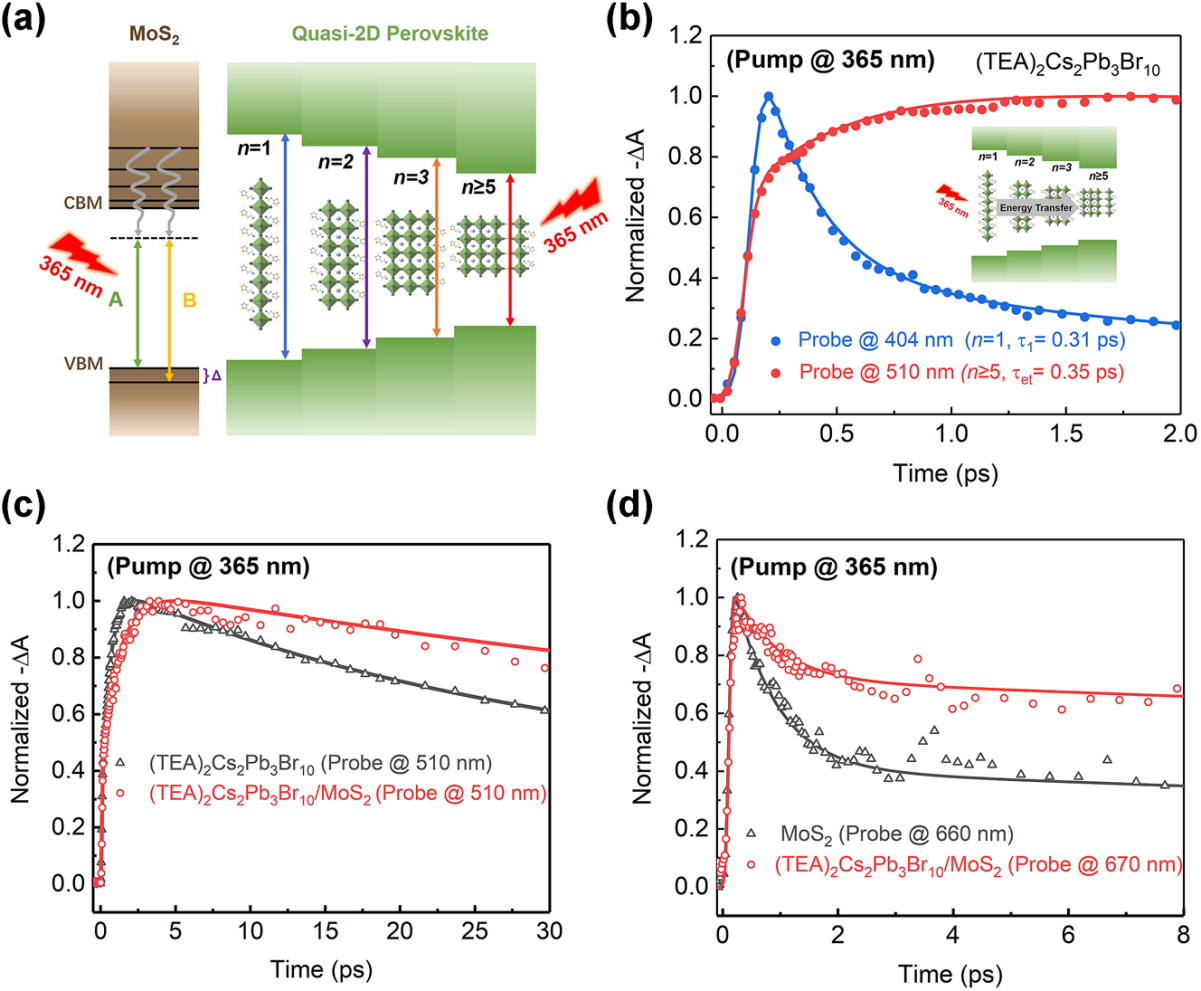
Dynamics of both hole transfer and electron transfer process. (a) Schematics of band structures of the heterostructure with (TEA)_2_Cs_2_Pb_3_Br_10_ quasi-2D RPP and MoS_2_ monolayer under 365 nm excitation. (b) TA kinetic curves at 404 nm and 510 nm for RPP. (c) TA kinetic curves at 510 nm for neat RPP and RPP/MoS_2_. (d) TA kinetic curves detected at 660 nm for neat MoS_2_ and heterostructure.

Figure 5a shows the schematic diagram that a 365 nm (3.40 eV) pump pulse with 45 μJ/cm^2^ fluence is used to excite both the MoS_2_ and (TEA)_2_Cs_2_Pb_3_Br_10_ in the heterostructure, and 510 nm (2.43 eV) and 660 nm (1.88 eV) light are adopted to probe (TEA)_2_Cs_2_Pb_3_Br_10_ excitons and MoS_2_ A-exciton, respectively (Figure 5a). In this case, the electrons (holes) in RPP (MoS_2_) are predicted to be transferred to the MoS_2_ (RPP) layer to form indirect excitons.

Different from the scenario with 510 nm excitation, the MoS_2_ and all-*n* phases in perovskites can be pumped with 365 nm wavelength. And photoinduced excited states or electrons with higher energy than that exited with lower energy photons are generated in both TMD and RPP. These hot electrons fall quickly to the CBM via nonradiative relaxation and engender intralayer excitons with holes in the corresponding VBM before disassociating into hot carriers in MoS_2_ and quasi-2D perovskites with different layers, respectively [55]. Figure 5b exhibits the GSB kinetic curves at 404 nm for *n* = 1 phase and 510 nm for *n* ≥ 5 phase in neat RPP under 365 nm excitation, respectively. The fitting result manifests that the transfer time of the electron carriers for the *n* = 1 phase is 0.31 ps, while it is 0.35 ps for *n* ≥ 5 quantum well, confirming the energy transfer from the small-*n* donor domain to the large-*n* acceptor domain.

The TA kinetics of *n* ≥ 5 phase in (TEA)_2_Cs_2_Pb_3_Br_10_ film and (TEA)_2_Cs_2_Pb_3_Br_10_/MoS_2_ heterostructure detected at 510 nm in 30 ps under 3.40 eV photoexcitation are compared in Figure 5c. The formation time is 1.58 ps and 3.9 ps for *n* ≥ 5 phase and the heterostructure, respectively, which suggests that there is a prominent hole transfer process in the heterostructure. The time constants *τ*_1_ and *τ*_2_ of 48.63 ps and 487.5 ps for the heterostructure, and 18.3 ps and 73.37 ps for the *n* ≥ 5 phase are obtained by fitting the decay signals with the triple-exponential equation, respectively. The slow decay constant of the perovskite layer in the heterostructure is almost 7 times that of the individual perovskite layer, indicating the transfer of holes from the MoS_2_ layer to the perovskite and the formation of interlayer exciton with a long lifetime.

We also extracted the dynamic curves probed at 660 nm and 670 nm for monolayer MoS_2_ and the heterostructure, respectively, shown in Figure 5d. In both samples, the signal reaches its maximum instantly upon excitation and follows a similar long-term biexponential decay trend after a fast decay process. The fast decay *τ*_1_ for the carrier cooling time and the slow decay component *τ*_2_ for the recombination time of the A-exciton in the heterostructure are 1.14 ps and 28.0 ps, larger than the respective constants of 0.58 ps and 5.13 ps in monolayer MoS_2_, respectively. This slow decay of the perovskite layer and the MoS_2_ layer indicates the formation of interlayer excitons, which is responsible for the recombination of separated charges in the heterostructure. Due to the slow recombination of the electrons from the MoS_2_ and the holes in the (TEA)_2_Cs_2_Pb_3_Br_10_, the exciton lifetime in heterostructure is longer than that in monolayer MoS_2_ or the quasi-2D RPP, respectively.

The faster-rising component probed at 660 nm in Figure 5d indicates that the electron-transfer time is shorter than the hole-transfer time from the rising component detected at 510 nm in Figure 5c for the heterostructure. The charge transfer rate in the heterostructure correlates with the positions of energy levels in the band alignment, temperature, mobility, and so on. A longer hole-transfer time is expected due to holes cascading from deeper valence states by defect engineering [56]. Furthermore, the holes localize in small-*n* domains in the presence of hole-trapping states and electrons in large-*n* domains [57]. Thus, the electron transfer in large-*n* domains is more energetically favorable and is faster, while the holes require more thermal assistance to transfer which slows down much more [58]. The decay is slower in the heterostructure regardless of which layer of the heterojunction is excited, verifying the long lifetime of the interlayer excitons due to the slow recombination rate. The longer charge-separated state provides promising results for photovoltaic and optoelectronic device applications.

## 3 Conclusions

2D (TEA)_2_Cs_2_Pb_3_Br_10_/MoS_2_ monolayer heterostructures were synthesized, and their photophysical properties were investigated by absorption, PL, TRPL, and TA spectra. The type-II band alignment at the heterostructure facilitates the separation of the photogenerated electron-hole pairs. TA measurements directly confirmed the excited holes in the MoS_2_ layer transfer across the interface to (TEA)_2_Cs_2_Pb_3_Br_10_ within 28.3 ps. And the excited electrons from (TEA)_2_Cs_2_Pb_3_Br_10_ transport to MoS_2_ rapidly in 0.12 ps. These results address the comprehension of the fundamentals between quasi-2D RPP and 2D MoS_2_ and would support the progress of optoelectronic.

## 4 Methods

(TEA)Br, CsBr, and PbBr_2_ were purchased from Xi’an Concentrator Technology Co., Ltd. The solvent dimethyl sulfone (DMSO, anhydrous, 99.999 %) was purchased from J & K Scientific. The materials were used directly without any further purification. Monolayer MoS_2_ films purchased from Six Carbon Inc. (Shenzhen, China) were deposited on sapphire substrates via the chemical vapor deposition technique.

According to the standard stoichiometric ratio of 2D RPP, (TEA)_2_Cs_2_Pb_3_Br_10_, (TEA)_2_PbBr_4_, and CsPbBr_3_ were fabricated by spin-coating a precursor solution of 2-TEA Bromide, CsBr, and PbBr_2_ dissolved in DMSO. The prepared solution was stirred at room temperature for 2 h (TEA)_2_Cs_2_Pb_3_Br_10_/MoS_2_, (TEA)_2_PbBr_4_/MoS_2_, and CsPbBr_3_/MoS_2_ vertical vdW heterostructures were fabricated by using the spin-coating method (4000 rpm, 50 s) and the annealing procedure (80 °C, 15 min).

The crystal structures and phases of (TEA)_2_Cs_2_Pb_3_Br_10_ film were characterized by using XRD, employing a scanning rate of 0.05° per second in a 2*θ* ranging from 2° to 50°, using a Bruker D8 Discover X-ray diffractometer with CuK_α_ radiation. Raman spectra were measured by LabRAM Odyssey Nano Laser enhanced *in-situ* sub-nano structure test spectrometer from HORIBA, France. The excitation wavelength is 532 nm. The steady-state absorption spectra of the quasi-2D perovskite film, monolayer MoS_2_, and the heterostructure were measured by UV–Vis spectrophotometer (Cary-60, Agilent). For the PL and TRPL measurements, samples were excited by a pulsed laser at 375 nm (> 28pJ/pulse), using the integrated fluorescence spectrometer (FluoroMax+, HORIBA) with a time-correlated single-photon counting (TCSPC) system. The ultimate temporal resolution of TRPL spectra is ∼25 ps.

The TA spectra were measured by using an fs pump-probe system consisting of the laser of a Ti: sapphire laser (Coherent, 800 nm, 35 fs, 7 mJ/pulse, and 1 kHz) and a spectrometer (Helios, Ultrafast Systems). The output laser light was split into two beams by using a separator. One output beam enters an optical parametric amplifier (OPA, 800 fs), which was mainly used to output an fs laser light. Here, 365 nm, 510 nm, and 660 nm pulses were used as the pump light to excite the samples. Another output light passes through a CaF_2_ crystal to form probe light from 320 nm to 680 nm. An optical delay-line kit was used to change the delay time between the pump and probe light. The pump light is cut off when it goes into the chopper before arriving at the sample. The sample was in excited states and not excited states alternately. A detector was used to record the relative intensity of [*I*(*λ*)_pro_/*I*(*λ*)_ref_]_pump_ and [*I*(*λ*)_pro_/*I*(*λ*)_ref_]_unpump_ of the probe pulse for the ‘excited’ and ‘unexcited’ sample by turns where *I*(*λ*)_ref_ is the optical intensity of the reference beam. The data were analyzed by Surface Xplore software and all measurements were performed at room temperature.

## Supplementary Material

Supplementary Material Details
