# Supplementary material for: Carrier transfer in quasi-2D perovskite/MoS2 monolayer heterostructure
Source: Nanophotonics. 2023 Nov 28;12(24):4495–505. doi: 10.1515/nanoph-2023-0570 (PMC11501760; doi:10.1515/nanoph-2023-0570)
Supplement: Supplementary file 1 — Supplementary Material Details [file j_nanoph-2023-0570_suppl_001.docx]

**Supplementary Material**

Carrier Transfer in Quasi-2D Perovskite/MoS_2_ Monolayer Heterostructure

Chaochao Qin^†^, Wenjing Wang^†^, Jian Song^†^, Zhaoyong Jiao^†^, Shuhong Ma^†^, Shuwen Zheng^†^, Jicai Zhang^†^, Guangrui Jia^†^, Yuhai Jiang^‡*^, Zhongpo Zhou^†*^

† Henan Key Laboratory of Infrared Materials & Spectrum Measures and Applications, and School of Physics, Henan Normal University, Xinxiang 453007, China

‡ School of Physical Science and Technology, ShanghaiTech University, Shanghai 201210, China

*Corresponding Authors:

Yuhai Jiang, e-mail: jiangyh3@shanghaitech.edu.cn;

Zhongpo Zhou, e-mail: zpzhou@htu.edu.cn


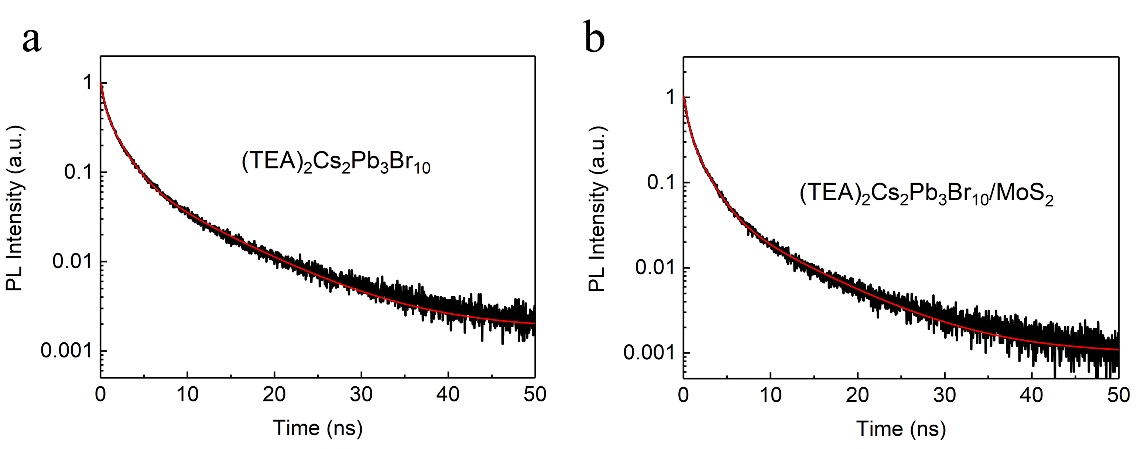


**Fig. S1**: Decay of the peak PL intensities for (a) (TEA)_2_Cs_2_Pb_3_Br_10_ and (b) (TEA)_2_Cs_2_Pb_3_Br_10_/MoS_2_ heterostructure, respectively. The PL was monitored at a 510 nm peak. Solid red lines are fits to a biexponential decay function.

**Tab. S1**. Fitting parameters of fluorescence lifetime for (TEA)_2_Cs_2_Pb_3_Br_10_ and (TEA)_2_Cs_2_Pb_3_Br_10_/MoS_2_ heterostructure with a bi-exponential function of $I\left( t \right)=A_{1}e^{(-t/\tau_{1})}+A_{2}e^{(-t/\tau_{2})}$ where A_i_ (%) and τ_i_ (ns) are the amplitude and time constant of the component i. The τ_avg_ represents the average fluorescence lifetime.

|  | **(TEA)_2_Cs_2_Pb_3_Br_10_** | **(TEA)_2_Cs_2_Pb_3_Br_10_/MoS_2_** |
| --- | --- | --- |
| A_1_ (%) | 70 | 73 |
| τ_1_ (ns) | 1.44 | 1.05 |
| A_2_ (%) | 30 | 27 |
| τ_2_ (ns) | 7.01 | 6.22 |
| τ_avg_ (ns) | 3.13 | 2.44 |


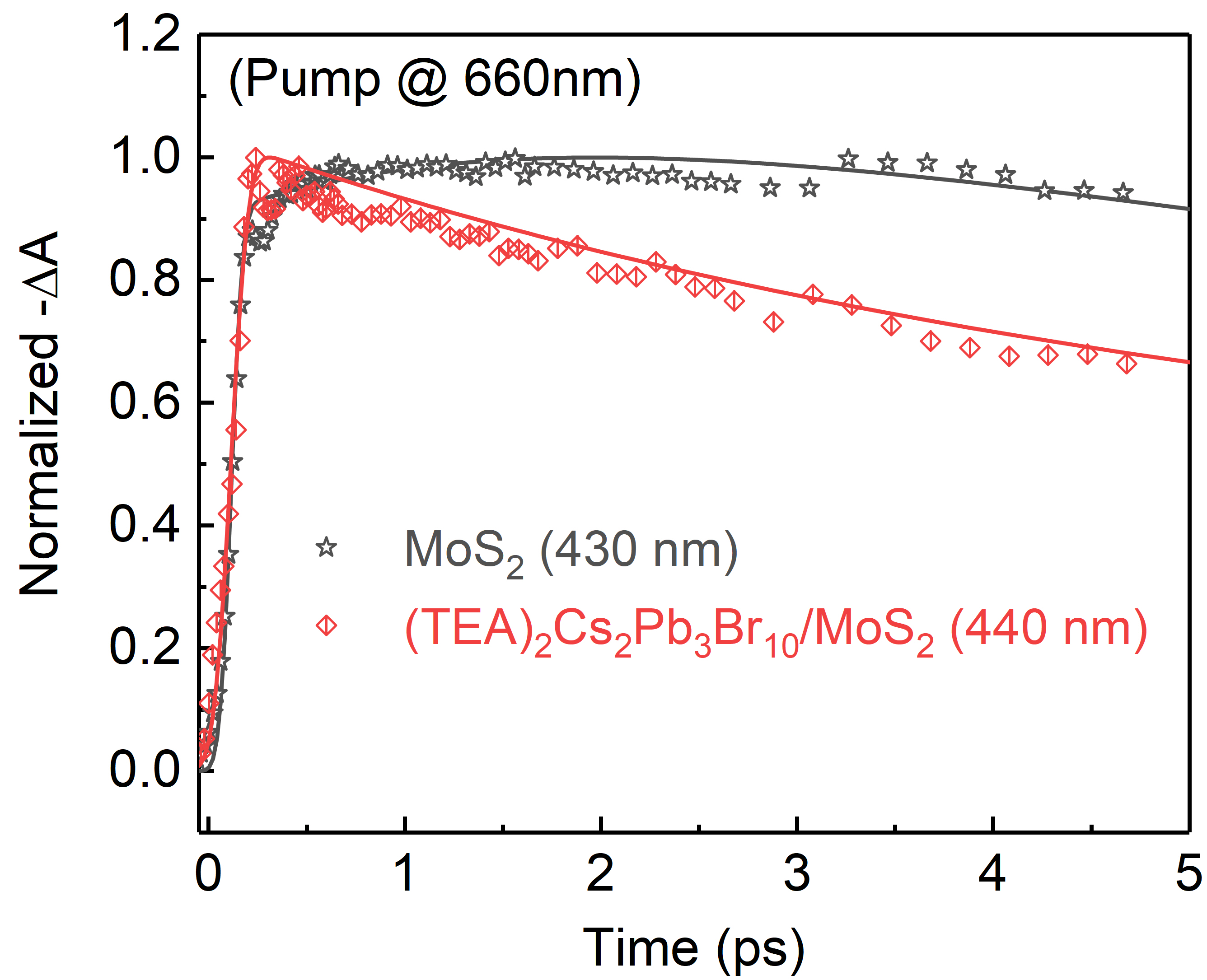


**Fig. S2**: TA kinetic curves probed at 430 nm and 440 nm for monolayer MoS_2_ and (TEA)_2_Cs_2_Pb_3_Br_10_/MoS_2_ heterostructure, respectively.


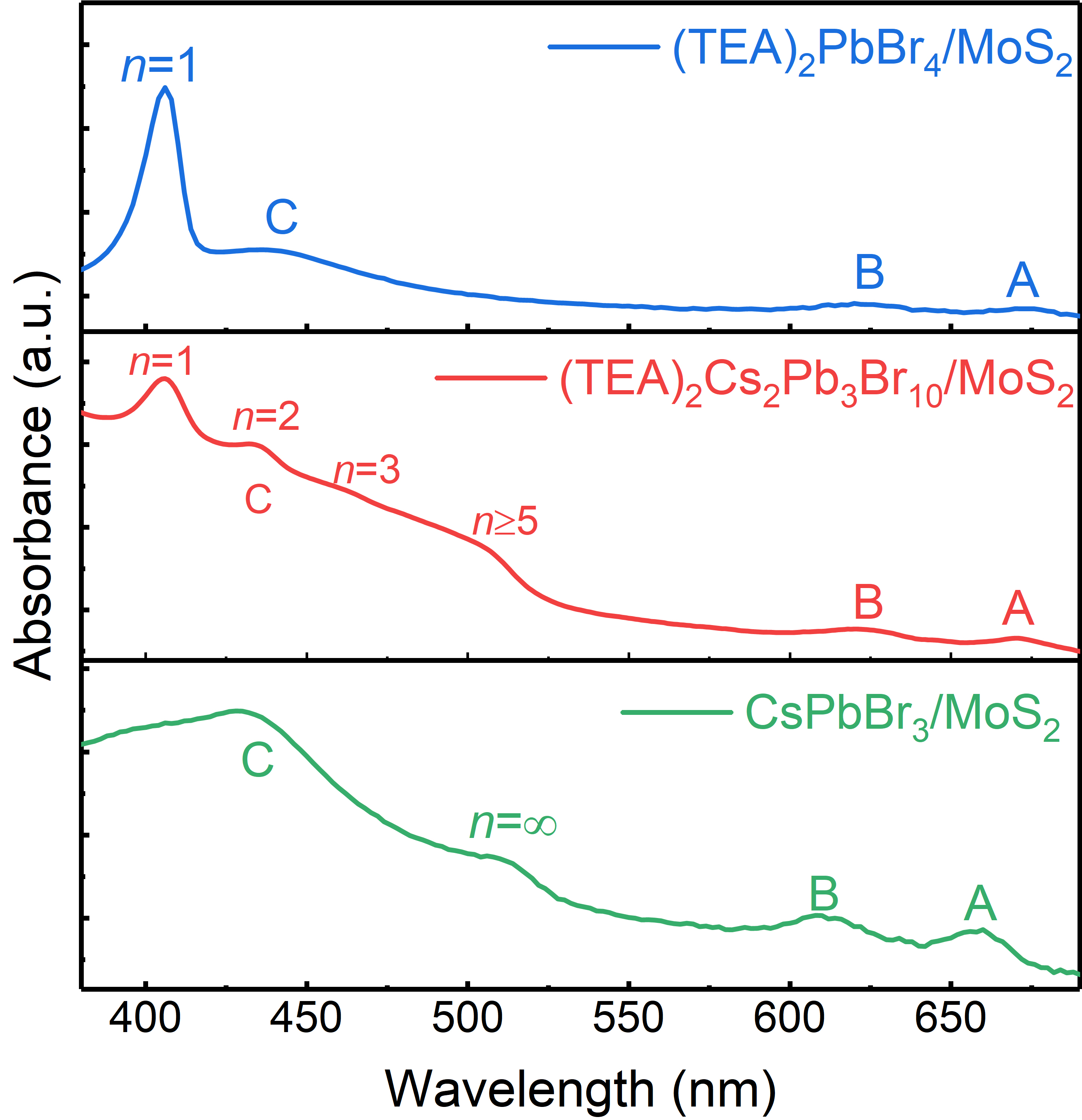


**Fig. S3:** Absorption spectra of (TEA)_2_PbBr_4_/MoS_2_, (TEA)_2_Cs_2_Pb_3_Br_10_/MoS_2_ and CsPbBr_3_/MoS_2_ heterostructures, respectively.

**Tab. S2:** Fitting parameters of TA kinetics of the B and C exciton by the equation (1) for MoS_2_ and (TEA)_2_Cs_2_Pb_3_Br_10_/MoS_2_ under 660 nm excitation. Parameters τ_1_ and τ_2_ are the time constants of the exponential components. Parameters a_1_ and a_2_ refer to normalized proportions.

|  | **MoS_2_**  **(B-exciton)** | **MoS_2_**  **(C-exciton)** | **(TEA)_2_Cs_2_Pb_3_Br_10_/MoS_2_**  **(B-exciton)** | **(TEA)_2_Cs_2_Pb_3_Br_10_/MoS_2_**  **(C-exciton)** |
| --- | --- | --- | --- | --- |
| a_1_ (%) | 22.4 | 67.9 | 54.1 | 34.4 |
| τ_1_ (ps) | 0.71 | 6.89 | 0.38 | 1.54 |
| a_2_ (%) | 79.8 | 32.1 | 45.9 | 66.6 |
| τ_2_ (ps) | 20.2 | 42.8 | 12.5 | 21.8 |

**Tab. S3.** Fitting parameters of TA kinetics of the B-exciton by the equation (1) for (TEA)_2_PbBr_4_/MoS_2_ and CsPbBr_3_/MoS_2_ under 660 nm excitation. Parameters τ_1_ and τ_2_ are the time constants of the exponential components. Parameters a_1_ and a_2_ refer to normalized proportions.

|  | **(TEA)_2_PbBr_4_/MoS_2_ (B-exciton)** | **CsPbBr_3_/MoS_2_ (B-exciton)** |
| --- | --- | --- |
| a_1_ (%) | 22.4 | 67.9 |
| τ_1_ (ps) | 0.71 | 0.43 |
| a_2_ (%) | 79.8 | 32.1 |
| τ_2_ (ps) | 20.2 | 18.4 |

**Tab. S4:** Fitting parameters of TA kinetics of the A and B excitons by the equation (1) in the case of MoS_2_ and (TEA)_2_Cs_2_Pb_3_Br_10_/MoS_2_ under 510 nm excitation. Parameters τ_1_ and τ_2_ denote decay time constants, and τ_et_ is the formation time constant. Parameters a_1_, a_2_, and c stand for amplitudes.

|  | **MoS_2_**  **(A-exciton)** | **MoS_2_**  **(B-exciton)** | **(TEA)_2_Cs_2_Pb_3_Br_10_/MoS_2_ (A-exciton)** | **(TEA)_2_Cs_2_Pb_3_Br_10_/MoS_2_ (B-exciton)** |
| --- | --- | --- | --- | --- |
| a_1_ (%) | 63.8 | 0.41 | 37.2 | 28.4 |
| τ_1_ (ps) | 0.26 | 0.58 | 1.08 | 0.82 |
| a_2_ (%) | 36.2 | 59.2 | 62.8 | 71.6 |
| τ_2_ (ps) | 6.92 | 13.0 | 13.7 | 27.3 |
| c (%) | 100 | 100 | 100 | 100 |
| τ_et_ (ps) | 0.09 | 0.10 | 0.12 | 0.11 |

**Tab. S5:** Fitting parameters of TA kinetics of the A-exciton by the equation (1) in the case of (TEA)_2_Cs_2_Pb_3_Br_10_, MoS_2_, and (TEA)_2_Cs_2_Pb_3_Br_10_/MoS_2_ under 365 nm excitation. Parameters τ_1_ and τ_2_ are the time constants of the exponential components. Parameters a_1_ and a_2_ refer to normalized proportions.

|  | **(TEA)_2_Cs_2_Pb_3_Br_10_**  **(*n*** ≥ **5)** | **MoS_2_**  **(A-exciton)** | **(TEA)_2_Cs_2_Pb_3_Br_10_/MoS_2_**  **(*n*** ≥ **5)** | **(TEA)_2_Cs_2_Pb_3_Br_10_/MoS_2_**  **(A-exciton)** |
| --- | --- | --- | --- | --- |
| a_1_ (%) | 45.4 | 77.0 | 50.9 | 71.1 |
| τ_1_ (ps) | 18.3 | 0.58 | 48.63 | 1.14 |
| a_2_ (%) | 54.6 | 23.0 | 49.1 | 28.9 |
| τ_2_ (ps) | 73.37 | 5.13 | 487.5 | 28.0 |
